# Tricyclic Antidepressants: Old Drugs-New Applications

**DOI:** 10.5812/kowsar.22287523.2100

**Published:** 2011-09-26

**Authors:** Nedim Solakovic

**Affiliations:** 1 Department of Anesthesiology, Reanimatology and Intensive Care, Cantonal Hospital Bihac, Bihac, Bosnia and Herzegovina

**Keywords:** Tricyclic antidepressants, Anesthetics, Amitriptyline, Doxepin

*Dear Editor*,

With great interest I have read an excellent article “Evaluation of the efficacy of intrathecal injection of amitriptyline and doxepin in spinal anesthesia in comparison with bupivacaine in rats“, published in your respected journal. The authors present and elaborate a very interesting subject of the application of tricyclic antidepressants as a local anesthetic. I totally agree with the authors of article that claim that there is a tendency of intrathecal injection of various drugs with different effect mechanisms (1). Several papers in the recent years, including the already mentioned article, indicate the local anesthetic effect of tricyclic antidepressants. In doing so, they demonstrate the effect of the drug on motor, sensory and proprioceptive fibers (2, 3). I think it would be very interesting to, by studying the above- mentioned block modes, to consider the possible effect of tricyclic antidepressants on the block of the vegetative (sympathetic) fibers and the possible hemodynamic changes that can occur. It is well known that in spinal anesthesia with conventional anesthetics, due to sympathetic blockade, very dramatic hemodynamic changes can arise, and that may be the cause of morbidity and mortality.

The article states that in group of tested animal to which amitriptyline were injected three rats died. The discussion states that the possible cause of death is heart failure due to the toxic effect of high-dosed amitriptyline. Unfortunately, drugs concentration in plasma in dead animals is unknown, as it was not the subject of the research. Is it possible that the amitriptyline cause extensive sympathetic block, which leads to a drastic reduction in cardiac output and peripheral vascular resistance and consequent cardiovascular failure in animals, rather than direct toxic effect?

Also, in future, if intention for studies in humans is planned, it should examine the neurotoxic potential of tricyclic antidepressants, especially in the intrathecal injection. For example, ten years ago, Gernes have presented a derivative of lidocaine, named tonicaine, with very strong local anesthetic properties. Block with tonicaine was four to nine times longer than with lidocaine; tonicaine had 80 times higher potential in blocking the Na+ currents than lidocaine, and possesed significantly better sensorimotoric discrimination than lidocaine. Unfortunately, due to evidence of neutotoxicity at lower concentrations, the researchers determined that the tested substance may have only limited clinical value (4).

In general, today's research into new intrathecal agents that could be used in spinal anesthesia and analgesia occurs in two directions: one is research of already well-defined substances for a new type of application-as intrathecal anesthetic or as an adjuvant (e. g. tricyclic antidepressants, midazolam, ά 2-agonists); the other direction is the exploration of entirely new substances. In this endeavor, a new synthetic peptide specifically for intrathecal use, ziconotide, has been introduced into clinical practice (USA 2004, EU 2005). It is intended for treatment of severe chronic pain who do not respond to or cannot tolerate opioid therapy (5).

The devolopment of the new anesthetic agents continues because it is relatively easy to synthetize substances with local anesthetic properties. Unfortunately, it is difficult to reduce the toxicity of these compounds because the most common side effects of local anesthetics present an extension of their therapeutic properties. New research on mechanisms of cardiac and spinal toxicity induced by the local anesthetic and identification of new drug target for spinal analgesia suggests the possible development of new (or old one for new type of application) and safer anesthetic in the future.
